# Orienteering experts report more proficient spatial processing and memory across adulthood

**DOI:** 10.1371/journal.pone.0280435

**Published:** 2023-01-20

**Authors:** Emma E. Waddington, Jennifer J. Heisz

**Affiliations:** Faculty of Science, Department of Kinesiology, McMaster University, Hamilton, ON, Canada; Technion Israel Institute of Technology, ISRAEL

## Abstract

The closest surrogate to hunter-gather activity is the sport of orienteering, which naturally and simultaneously combines high-intensity interval exercise with navigation. Although human cognition can be improved across the lifespan through exercise and cognitive training, interventions like orienteering may be especially effective because they resemble activities engaged in by prehistoric humans during evolution. The present study tested whether orienteering experts have better hippocampal-dependent cognitive function than active, non-orienteering controls. One-hundred and fifty-eight healthy adults between the ages of 18 and 87 years old with varying experience in orienteering (none, intermediate, advanced, elite) reported on their spatial processing, spatial memory and episodic memory using the Navigational Strategy Questionnaire and the Survey of Autobiographical Memory. Orienteering experts reported greater use of allocentric and egocentric spatial processing and better spatial memory than controls. In contrast, episodic memory was not associated with orienteering expertise. Notably, the significant effects of orienteering on spatial cognition remained even after controlling for age, sex, and physical activity, suggesting that orienteering may be an effective intervention to prevent age-related cognitive decline in spatial navigation and memory.

## Introduction

The human brain evolved under the harsh environmental conditions of our evolutionary past, and modern life may lack the specific cognitive and physical challenges that the brain needs to thrive [1,2]. Consider navigation, for example; humans are no longer required to hunt and gather food to survive and now rely on passive transport and Global Positioning System (GPS) navigation [3–5]. While GPS navigation is convenient, it requires minimal cognitive stimulation, and its long-term use can impair spatial memory skills during self-guided navigation tasks such as map reading [3]. Without consistent use of our navigational aptness, we may risk losing aspects of our spatial processing abilities and their supporting neural architecture. Consequently, this could impact various aspects of human cognition [2,6].

In healthy aging, there is unequal atrophy across the human brain. The hippocampus atrophies more than the parietal regions [7], impairing memory and particular spatial processing abilities more than others [8]. For example, allocentric spatial processing, a navigational strategy from an object-based or third-person perspective (relative to the environment), is mediated by the hippocampus and is more impaired with advancing age [8,9]. In contrast, egocentric spatial processing, a navigational strategy based on a first-person perspective (relative to the body), is mediated by the caudate nucleus and parietal regions and is less impaired with advancing age [8,10]. Successful navigation requires flexibly switching and combining these different spatial strategies. Here, spatial information is translated from its stored form in an allocentric reference frame into an egocentric reference frame to use while navigating [11,12]. However, the age-related losses in allocentric processing lead older adults to rely more on egocentric processing [13,14]. Wayfinding can also be achieved using a route or procedural strategy, a less robust, non-spatial navigation technique that relies on memorizing a list of landmarks as cues for turning directions [15] and can be compromised by aging [16]. Taken together, older adults tend to experience more navigational problems that may ultimately limit their mobility [17].

Compared to healthy aging, age-related impairments in general cognition, such as mild cognitive impairment and Alzheimer’s disease, are accompanied by more significant hippocampal degeneration [18,19], further impairing their allocentric spatial processing and wayfinding abilities [20,21]. At worse, the individual may be unable to create, store or use mental maps of new environments needed to navigate [22–24], a multifaceted condition known as topographical disorientation [25,26]. Even self-reported complaints about spatial navigation abilities are higher in those with mild cognitive impairments and Alzheimer’s disease than controls [27]. Evidence suggests that early signs of topographical disorientation may be a predictor of general cognitive impairment later in life, whereby healthy middle-aged adults with risk factors for dementia also tend to have poorer allocentric spatial processing than their peers [21]. It follows that interventions designed to strengthen allocentric spatial processing may also help stave off general cognitive decline and topographical disorientation [28].

According to the Adaptive Capacity Model [2], coupling navigation-based tasks with movement may be an effective mechanism to enhance cognition in domains that often deteriorate with age. The model theorizes that the human brain may be more receptive to interventions that resemble the hunter-gatherer activities that humans engaged in when the human brain evolved, such as memorizing spatial routes while navigating [2,29]. Although physical exercise alone increases cognition [30–32] and neurogenesis [33–36] and can add to the benefits of cognitive training [37–41], many interventions have participants perform exercise training and cognitive training asynchronously. This separation may, however, lessen the evolutionary semblance of the training and therefore diminish the associated cognitive gains. Indeed, a recent meta-analysis found synchronous training to be slightly (though not significantly) more effective at improving cognition in older adults than sequential training [42]. We propose that the effect size of synchronous over asynchronous training may depend on how seamlessly the cognitive and exercise training components are combined.

The sport of orienteering seamlessly and simultaneously integrates movement with navigation that is prehistorically familiar to the brain and, therefore, is the closest surrogate to hunter-gatherer activities [2]. The goal of orienteering is to navigate by running as quickly as possible over unfamiliar terrain through a series of checkpoints using only a topographical map and a compass [43]. The most skillful orienteers must efficiently switch between various mental tasks, making quick decisions while moving across the terrain at a rapid pace [44,45]. The unique thing about orienteering is that it requires active navigation, encompassing quick and recurrent transitions between allocentric and egocentric spatial processing in real-time, which GPS systems have engineered out of modern life [3,6]. The frequent and intermittent engagement of allocentric processing by orienteers may help them counteract the typical decline that occurs with aging. However, little research has assessed the effects of real-world orienteering experience on hippocampal-related functioning. One study found that expert orienteers with over eight years of experience in the sport outperformed non-orienteering controls on tasks involving object-based mental rotation [46]. Another research group had 15 students complete VR orienteering races over six days and observed improvements in the same object-based mental rotation task but did not compare the effects to real-world orienteering experience [47]. They also failed to include the physical stimulus of exercise, which we would argue makes orienteering so unique to the field of exercise cognition. A third study had 24 college students complete a 12-week real-world orienteering intervention at a moderate exercise intensity and observed improvements on a computer-based two-dimensional visuospatial working memory task, however, the improvements were only observed in male participants [48]. While these findings are promising, more work is needed.

The present study examined the effects of orienteering, which simultaneously combines movement and navigation, on hippocampal-related functioning in adults aged 18 to 87 years old with varying orienteering skill levels (intermediate, advanced, elite) plus a physically active non-orienteer control group. We included a physically active control group who had little to no orienteering experience to assess the effect of orienteering expertise over and above the benefits of physical activity engagement without navigation. All participants reported on their spatial processing, spatial memory and episodic memory using the Navigational Strategy Questionnaire and the Survey of Autobiographical Memory. We expected participants with more orienteering experience to report greater use of hippocampal-related allocentric spatial processing. We also examined whether orienteering experience would transfer to other spatial and episodic memory domains that also depend on the hippocampus. Finally, we examined whether the benefits of orienteering on spatial processing and memory remained after controlling for the effects of age, sex, and physical activity level.

## Methods

### Participants

One hundred and fifty-eight healthy adults (77 men, 81 women; age *M (SD) =* 48 (± 20) years, range = 18–87 years) completed the online survey. Orienteers (N = 114) of subjectively rated intermediate to elite skill level who regularly participate in the sport were recruited from orienteering clubs from around the world using the following inclusion criteria: English language proficiency, participation in foot-orienteering five times or more from intermediate to elite skill level, not exceeding moderate weekly alcohol and/or drug use (≤ 10 servings of alcohol per week [49], ≤ 7 days per week using electronic or regular cigarettes, and infrequent (weekly or less) use of marijuana [50] or other recreational drugs) and having no diagnosis of mild cognitive impairment. To assess the effect of orienteering compared to other forms of aerobic exercise that are less cognitively and navigationally demanding, physically active individuals (N = 44) from aerobic-based sports organizations (i.e., running, cycling, etc.) from around the world were recruited into the control group using the same inclusion criteria. Considering previous work that grouped participants as “orienteers” if they had a minimum of six 2-hour sessions of formal orienteering training [51], control participants in this study were included if they had either no orienteering experience (zero exposures) or an insignificant level of exposure that would not impact their navigational aptitudes (i.e., four or fewer single-session exposures).

All participants provided written informed consent through the online questionnaire and were able to enter a draw to receive an honorarium of fifty Canadian dollars. This project was approved by the McMaster Research Ethics Board (# 2394).

### Procedure

All participants completed an online questionnaire (LimeSurvey Software) consisting of a demographic questionnaire, the Navigation Strategy questionnaire [15], and the Survey of Autobiographical Memory [52], outlined below.

Participants in the orienteering group completed additional questions based on their orienteering experience, which consisted of a series of evidence-based questions created ad hoc through consultation with orienteering experts to assess common orienteering concepts (see S1 Appendix) [45]. Orienteering skill can be ascertained by the difficulty of courses typically navigated and the frequency and magnitude of deviations from the optimal or intended route. In orienteering, these deviations are referred to as “mistakes”, which become smaller and less frequent with increasing skill levels [45]. As such, responses from the second question of this survey were used to group orienteers into the appropriate skill level (i.e., *intermediate*: “I can navigate well both on and off trails but often have large issues”; *advanced*: “I can navigate well off trails with few available features and seldom make large mistakes”; *elite*: “I hardly ever make errors in my navigation and feel confident in many kinds of terrain”) whereby those with greater skill level also tended to have reported a greater number of years of orienteering experience.

#### Demographic information

Demographic information, including the year of birth, sex, nationality, employment status, and education, was collected. All participants completed the Physical Activity and Sedentary Behaviour Questionnaire (PASB-Q) from the Canadian Society for Exercise Physiology [53]. Total aerobic physical activity from the PASB-Q was used to estimate the participant’s weekly exercise engagement by multiplying the average number of active days per week by the average length of active time (minutes/week).

#### The navigational strategy questionnaire

The Navigational Strategy Questionnaire (NSQ) [15] was used to evaluate the navigational tendencies of all participants. In the NSQ, participants used a 5-point Likert scale to rank 44 items which described concepts related to one of three navigational strategies to receive an average score for each strategy. The use of hippocampal-dependent allocentric spatial processing was assessed with questions such as “I usually attempt to mentally represent route segments, turns and their spatial relationships from a top-down aerial perspective” (Question 14, NSQ). The use of egocentric spatial processing indicates that individuals approach navigational tasks from a first-person view and can: “… visualize [the] environment in the form of a 3D spatial layout …” (Question 31, NSQ). Though egocentric navigation is not dependent on hippocampal processing [10], the hippocampus aids in transitioning between egocentric and allocentric spatial processing when navigating. The use of the procedural strategy assesses an individual’s reliance on non-spatial methods for navigation with a preference for: “…following directions with descriptions of landmarks at turning points rather than using a map” (Question 18, NSQ).

#### The survey of autobiographical memory

The Survey of Autobiographical Memory (SAM) Questionnaire is a 26-item subjective memory survey that gives a general self-reported measure of episodic, semantic, spatial, and future memory [52]. Each item is answered using a five-point Likert scale, and scores are weighted appropriately and summed for each memory type. In this project, we analyzed spatial and episodic memory, with statements such as: “In general, my ability to navigate is better than most of my family/friends” (Question 1, SAM) for spatial memory and “When I remember events, I remember a lot of details” (Question 6, SAM) for episodic memory.

### Statistical analysis

All data were analyzed using SPSS (IBM SPSS Statistics for Macintosh, version 28.0; IBM Corp., Armonk, NY). Raw data from the online questionnaire software were screened for missing cells (unanswered questions). Normality was assessed using the Shapiro-Wilk test and using visual inspection of histograms. Not all variables showed normal distribution, but all showed strong linearity when examined visually using normal Q-Q plots. As such, non-parametric tests were used.

Kruskal-Wallis H tests were used to compare mean rank differences between the level of orienteering experience with demographic factors (age, physical activity, sex, years of orienteering experience) and cognition (allocentric spatial processing, egocentric spatial processing, procedural navigation, spatial memory, episodic memory). Post hoc tests were completed using Dunn’s pairwise tests for all four pairs of orienteering experience level groups. Spearman’s rank correlations were also used to assess the associations of orienteering experience with demographic factors and cognition, and partial Spearman’s correlations were used to evaluate whether the relationships between orienteering experience and cognition remained after controlling for age, sex, and physical activity. All statistical tests were computed using a 95% confidence interval and an alpha criterion of 0.05.

## Results

### Participants

Participant demographics by group can be found in Table 1. Elite orienteers were younger in age (*H*(3) = 15.66, *p* = .001; ε^2^ = .10) and more physically active (*H*(3) = 14.59, *p* = .002; ε^2^ = .09) than intermediate or advanced orienteers but only differed from controls in physical activity levels (*p* < .05). The sex of participants differed between groups (*H*(3) = 13.41, *p* = .004; ε^2^ = .09), with a higher male-female ratio among elite orienteers than intermediates or controls (*p* < .05). Although education level was relatively balanced across the groups, especially in post-secondary and post-graduate studies, control participants differed compared to elite and advanced orienteers and elites differed compared to intermediates (*p* < .05). Subjectively rated orienteering level was significantly associated with the number of years of orienteering experience (*r*_*s*_(158) = .78, *p* < .001), where 47% (N = 34/72) of advanced and elite orienteers combined reported 20 or more years of experience.

**Table 1:** Descriptive statistics and average outcome measures across groups.

	*Control*	*Intermediate*	*Advanced*	*Elite*
*n*	44	42	41	31
Age (years)	48.4 ± 21.7	54.4 ± 18.1	51.5 ± 20.4	35.3 ±15.1
Age range (years)	19–86	22–87	18–84	20–66
Sex (F/M)	27/17	28/14	17/24	9/22
Physical Activity (minutes/week)	360 ± 335	238 ± 142	274 ±175	423 ± 207
Nationality (%) North America Europe Oceania Asia	84% 9% 0% 7%	90% 10% 0% 0%	71% 29% 0% 0%	29% 68% 3% 0%
Education (%) < Secondary Secondary Post-Secondary Post-Graduate Did not answer	0% 27% 43% 27% 2%	0% 7% 40% 52% 0%	2% 15% 37% 44% 2%	0% 29% 35% 35% 0%
Employment Status (%) Unemployed Student Employed *	2% 25% 73%	0% 5% 95%	0% 20% 80%	0% 39% 61%
Mode Range in Years of Orienteering Experience	N/A	6–10	20+	20+
NSQ Egocentric	3.1 ± 0.8	3.0 ± 0.7	3.7 ± 0.5	3.6 ± 0.6
NSQ Allocentric	3.2 ± 0.6	3.1 ± 0.6	3.6 ± 0.5	3.5 ± 0.6
NSQ Procedural	3.7 ± 0.5	3.4 ± 0.5	3.1 ± 0.5	2.8 ± 0.5
SAM Spatial	98.0 ± 12.2	98.5 ± 12.3	110.8 ± 9.6	112.2 ± 9.5
SAM Episodic	100.6 ± 12.6	96.2 ± 11.4	98.7 ± 15.4	97.1 ± 13.5

NSQ, Navigational Strategy Questionnaire; SAM, Survey of Autobiographical Memory. *Employed includes full-time (>40 hours/week), part-time (<40 hours/week), self-employed, or retired.

Values represent Mean ± SD.

### Egocentric, allocentric and procedural spatial processing

**Fig 1:**
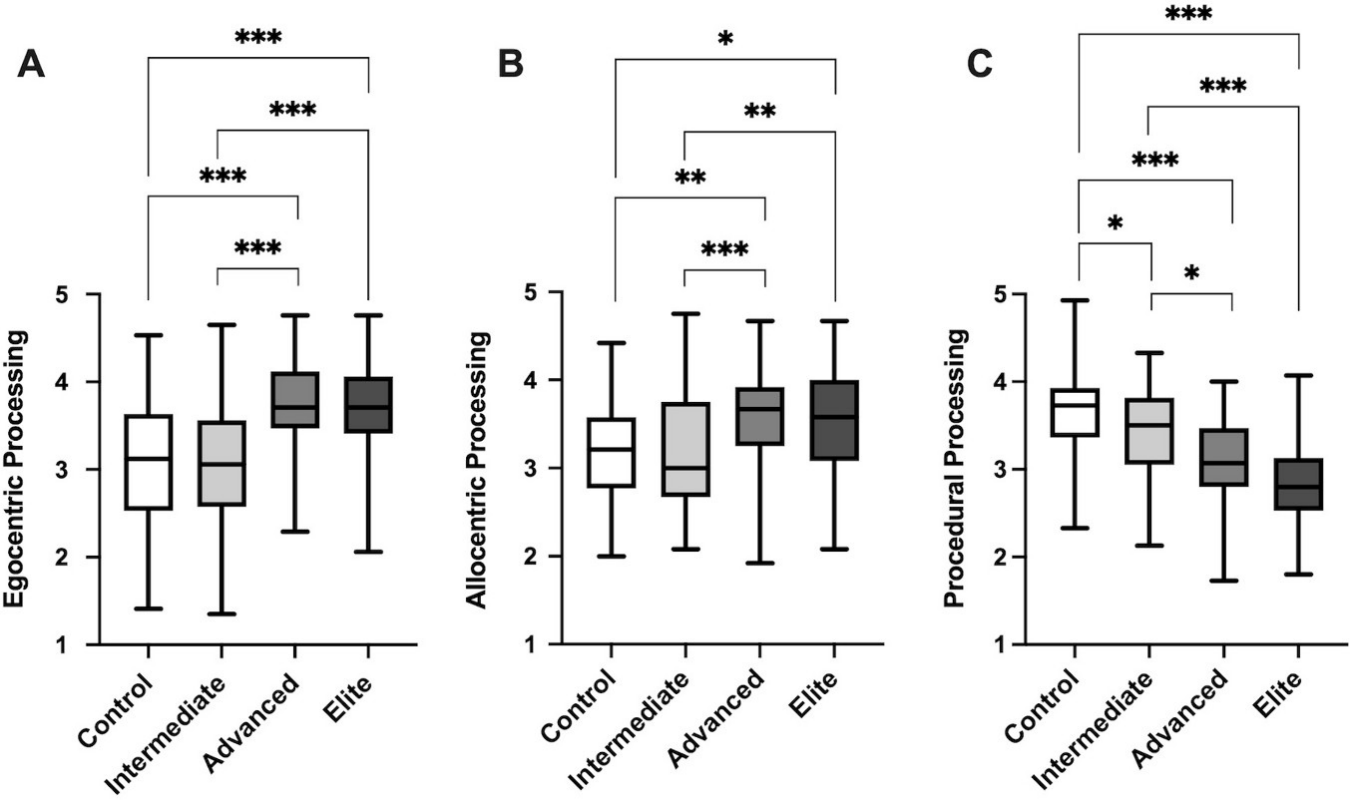
Average score on NSQ spatial processing measure across groups. A boxplot demonstrating mean, minimum, and max values for A) egocentric spatial processing, B) allocentric spatial processing, and C) procedural processing. Error bars reflect SD.* reflects *p* < .05, ** reflects *p* < .01, *** reflects *p* < .001.

Fig 1 depicts the large positive effect of orienteering on self-reported egocentric processing (*H*(3) = 31.16, *p* < .001; ε^2^ = .20), allocentric processing (*H*(3) = 17.98, *p* < .001; ε^2^ = .11), as well as the negative effect of orienteering on procedural navigation (*H*(3) = 44.10, *p* < .001; ε^2^ = .28). Post hoc tests using Dunn’s pairwise test indicated that advanced and elite orienteers reported engaging in more allocentric and egocentric processing, and less procedural processing than controls or intermediate participants (all *p* < .05). There were no differences between control and intermediate, or between advanced and elite orienteers for either allocentric or egocentric spatial processing (*p* > .05); however, there was a difference between intermediate orienteers and controls for procedural processing, where intermediates indicated less reliance on this strategy (*p* < .05).

### Spatial and episodic memory

**Fig 2:**
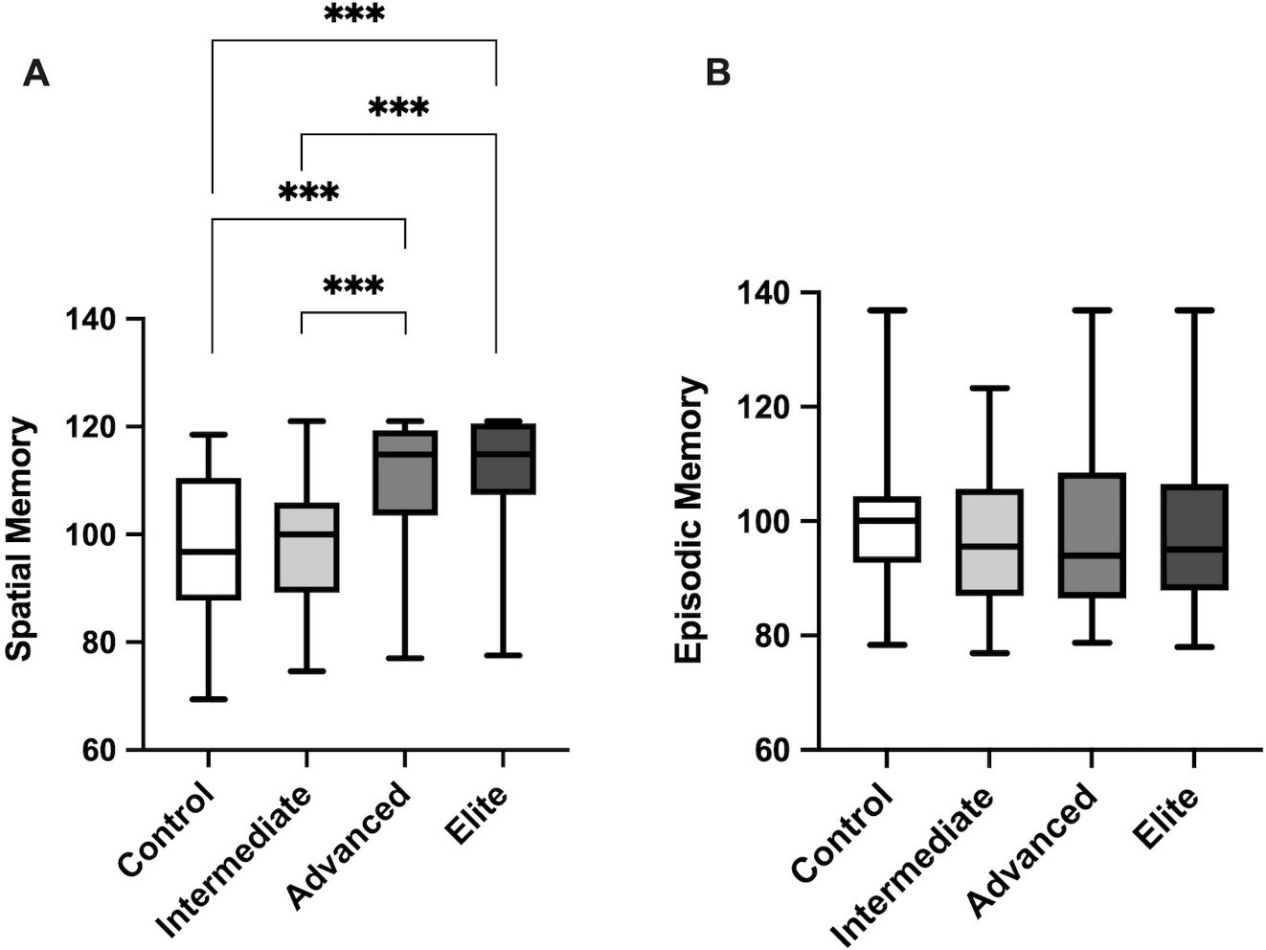
Average score on SAM autobiographical memory measure across groups. A boxplot demonstrating mean, minimum, and max values for A) spatial memory, B) episodic memory. Error bars reflect SD. *** reflects *p* < .001.

Fig 2 depicts the large effect of orienteering on self-reported spatial memory (*H*(3) = 45.51, *p* < .001; ε^2^ = .29). Elite and advanced orienteers reported better spatial memory than intermediate orienteers and non-orienteers. Post hoc tests using Dunn’s pairwise tests indicated that advanced and elite orienteers had greater ratings of spatial memory than either control or intermediate orienteers (*p <* .001). There was no difference between advanced and elite or between control and intermediate participants (*p* > .05). In contrast, orienteering experience was not associated with self-reported episodic memory (*H* (3) = 2.75, *p* = .433; ε^2^ = .02) at any group level.

### Covariates of age, sex, and physical activity

Consistent with the Kruskal-Wallis H tests, we observed significant Spearman rank correlates, revealing that greater orienteering experience was positively associated with egocentric spatial processing (*r*_*s*_(158) = .38, *p* < .001), allocentric spatial processing (*r*_*s*_(158) = .28, *p* < .001) and spatial memory (*r*_*s*_(158) = .50 *p* < .001) but negatively associated with procedural navigation (*r*_*s*_(158) = -.53, *p* < .001). Participants with greater orienteering experience also tended to be younger (*r*_*s*_(158) = -.17, *p* = .038), male (*r*_*s*_(158) = -.26, *p* < .001), and more physically active (*r*_*s*_(158) = .17, *p* = .033); however, entering age, sex, and physical activity as covariates in the partial correlation analyses did not alter the associations between orienteering experience, spatial processing, or memory. Table 2 shows the correlational matrix of the relationships between orienteering experience with all facets of the NSQ and SAM while also controlling for age, sex, and weekly physical activity level.

**Table 2:** Correlation matrix with age, sex, and weekly physical activity as control variables.

Control Variables		1	2	3	4	5	6
**Age, Sex, & Weekly Physical Activity**	**1. Orienteering Experience Level**	-					
	**2. NSQ Egocentric**	.30 ***	-				
	**3. NSQ Allocentric**	.22 **	.45 ***	-			
	**4. NSQ Procedural**	-.47 ***	-.37 ***	-.37 ***	-		
	**5. SAM Episodic**	-.05	.26 **	-.03	.12	-	
	**6. SAM Spatial**	.41 ***	.72 ***	.40 ***	-.43 ***	.18	-

NSQ, Navigational Strategy Questionnaire; SAM, Survey of Autobiographical Memory.

** reflects *p* < .01,

*** reflects *p* < .001.

## Discussion

The present study examined whether more experience in the sport of orienteering was associated with greater self-reported use of hippocampal-dependent spatial processing tendencies. Orienteers were expected to have higher self-reported allocentric spatial processing abilities because the sport encompasses the utilization of this process more than in modern life. Interestingly, elite and advanced orienteers reported greater use of allocentric and egocentric spatial processing and had higher subjective spatial memory than physically active controls with little to no orienteering experience. The results remained significant even after controlling for age, sex, and physical activity, suggesting that engagement in the sport of orienteering may be beneficial to essential aspects of spatial processing and memory across the lifespan.

The observed association between orienteering and spatial processing is particularly impressive given that the age range of our sample was 18 to 87 years old, and the end of this age range is typically marred by marked declines in allocentric processing [8]. Of course, this is a cross-sectional study, and we must include the fact that those with better spatial processing may be merely self-selecting into the sport. At least one study found evidence for trait-like differences in expert orienteers who reported experiencing more pleasure from exploring novel terrains than beginner orienteers or controls [46], and 60% of the orienteers in this study indicated monthly usage of a map and compass outside of the sport of orienteering (see S1 Table). Yet, the alternative is worth considering—that the engagement in orienteering may be neural protective to spatial domains of cognition, including both spatial processing and spatial memory. This interpretation is consistent with prior work demonstrating the effectiveness of short-term orienteering training on visuospatial working memory [48] and longitudinal data demonstrating that greater GPS use is associated with a steeper decline in hippocampal-dependent spatial memory [3]. Taken together, our results and others suggest that aging-related decline in spatial memory may not only be related to aging per se but also to losing what we do not use (i.e., “use it or lose it”). Unlike most modern-day dwellers, orienteers regularly engage in active navigation, which may mitigate their cognitive decline as they age.

The Adaptive Capacity Model would attribute the large benefit of orienteering on cognition to the evolutionary semblance of the sport and the brain’s receptiveness to such activities [2]. The quick and recurrent transitions between allocentric and egocentric spatial processing that orienteers engage in may also play an important role. An orienteer’s successful navigation and route recall depend on their ability to translate and integrate allocentric and egocentric representations while synchronously navigating and moving throughout the surrounding environment [11,12]. The routine use of this allocentric-to-egocentric spatial translation could explain why the orienteers perceived their abilities to be superior in hippocampal-dependent allocentric spatial processing, egocentric spatial processing, and spatial memory. In the context of aging-related changes, spatial memory is hypothesized to provide a “scaffold” for episodic memories [54], and the inability to reinstate a spatial scaffold from long-term memory is postulated to underlie memory impairments in Alzheimer’s disease [55]. Given that engagement in the sport of orienteering was associated with better spatial memory reports across the lifespan, it follows that orienteering may be a viable intervention strategy to reduce the risk of Alzheimer’s disease.

That said, orienteers did not rate episodic memory differently relative to the active control group. This may be surprising given that spatial memory provides crucial information for episodic memory concerning “where” the episode (or event) took place. However, several things may account for this. First, the control group was very physically active, and physical activity, especially when done at a high intensity, enhances episodic memory across the lifespan [38,56]. While the orienteers in our sample were frequently physically active, it is possible that their activity was not vigorous enough to facilitate neurogenic adaptations needed to enhance episodic memory. To further investigate the impacts of orienteering on episodic memory, future work should include measurements of exercise intensity or compare orienteers to a sedentary control group, which is minimally examined in the current literature.

Alternatively, adding navigation to physical activity may not boost episodic memory over and above the benefits of physical activity alone. Episodic memory can rely on non-spatial elements, including people and objects [54], and the orienteers were less likely to use non-spatial procedural navigation strategies. This may suggest a potential tradeoff where orienteers favour spatial over non-spatial information processing resulting in no net gain for episodic memory.

With respect to orienteering skills, elite and advanced orienteers perceived their spatial cognition to be superior to that of intermediate orienteers even after controlling for age, sex, and physical activity, though advanced and elite groups did not often differ. This can be seen in the positive association between higher orienteering skill and the number of years of experience, where almost half of the advanced and elite orienteers indicated 20 or more years of experience, whereas the majority of intermediate orienteers reported less than 20 years of experience (S1 Table). Most advanced and elite orienteers also reported training and racing more frequently in orienteering compared to the intermediates (S1 Table). These results suggest that prolonged and consistent practice of orienteering is required for cognitive gains. Another point to consider is that a high proportion of elite orienteers in our sample were European. Although people living in Europe tend to have relatively good navigational abilities compared to other countries across the globe, this may be especially true in countries like Finland, Norway, and Sweden because they include orienteering in school curriculums and tend to have higher success rates at international competitions [57]. However, in the context of “use it or lose it,” even active rehearsal of lower-level orienteering skills would be expected to strengthen unused neural pathways. Ideally, we would have recruited beginner-level orienteers to test this hypothesis; however, none of our participants were at that level. Nonetheless, the “use it or lose it” hypothesis is supported by the prior observations that short-term real-world orienteering training results in spatial processing gains [48] and that individuals who rely less on GPS have better navigation skills [3]. Longitudinal interventions are needed to determine how much navigational practice is required to alter evolutionarily relevant but neglected navigational skills.

While this study makes an important contribution to understanding the benefit of orienteering for human cognition, it is not without its limitations. This virtual study allowed us to recruit a large and diverse sample of participants from across the globe, and we used self-reported assessments to explore multiple cognitive domains relating to hippocampal functioning in a quick and accessible way. Although self-reports do not always correspond well to objective measures of cognition [58,59], physical tasks tend to be less impacted by metacognitive inaccuracies than cognitive tasks [60] and the self-reported assessments used here show good corresponded with objective measures [15,52,61]. Furthermore, when it comes to Alzheimer’s and related dementias, subjective memory complaints are often used as an early indicator of increased risk, even in the absence of overt memory impairment [27]. That said, as future research continues to examine how orienteering expertise impacts objective cognitive performance, our self-reported results suggest that certain cognitive domains, namely, spatial navigational strategies and spatial memory, should be further compared with objective measures.

As the research on orienteering for cognition evolves, it will be necessary to operationally define what it means to be an “orienteer” and what factors should be examined when classifying one’s level of orienteering expertise. In this study, we used an *ad hoc* questionnaire asking participants to report on their orienteering experience (see S1 Appendix). Although our classification of orienteering expertise was subjective, the self-reported rankings correlated well with the participants’ perceived skill level and years of orienteering experience. Based on prior literature, the least amount of experience needed to qualify as an “orienteer” was six to eight 2-hour formal orienteering training sessions [51]. Another prior study used the criteria of “more than five years of experience” to classify beginner orienteers [46]. Most of the orienteers in this study fell beyond this criterion, with more than one-third of participants reporting over 20 years of experience. Moreover, although we included sex as a covariate in our analyses and it did not impact our results, males and females often approach navigational tasks differently [62], and one prior study of 24 participants found that only males benefited from a 12-week orienteering training program whereas females did not [48]. Moving forward, it is important for future research to consider sex differences in the effects of orienteering on cognition.

Finally, although we controlled for many factors, we did not control for the participants’ prior experience with VR or video gaming. One question (See S1 Appendix: Q5) evaluated the orienteers’ weekly use of orienteering maps without being physically active (i.e., map making or playing orienteering virtual reality (VR) videogames), and only 21% of the orienteers (N = 24/114) engaged in these tasks for more than 1 hour per week (S1 Table). To date, many interventions designed to boost navigational abilities have relied on navigational training using VR [24,63]. For example, a recent study found that playing a 3D navigation-based videogame for five consecutive days improved both allocentric spatial processing and spatial memory [63]. However, based on our understanding of the Adaptive Capacity Model [2], coupling VR training with movement may replicate real-world navigation and provide greater cognitive benefits. This is supported by two studies that found that when coupling VR spatial navigation training with treadmill walking over four months, expected age-related atrophy of the hippocampus could be mitigated [64] and decreases in cortical thickness were prevented [65]. Given the promising effects of VR navigation training on cognition, one would expect the cognitive benefits to be augmented by real-world navigation training, such as orienteering. Future work related to orienteering should compare real-world navigation with VR navigation while also controlling for other factors that may affect spatial processing capacity, including video game use, type and cognitive demand of occupation, and sports engagement.

## Conclusion

In conclusion, expert orienteers reported greater reliance on allocentric and egocentric spatial processing abilities and had better perceived spatial memory capacity. This is the first study to demonstrate a link between engagement in the sport of orienteering and hippocampal-dependent processes, and it suggests that training in the sport of orienteering may be more beneficial than physical activity alone. Although this cross-sectional study cannot speak to the causality of these associations, these novel results point to the sport of orienteering as a viable brain training regime to combat age-related cognitive decline.

## Supporting information

S1 Appendix Orienteering experience questionnaire. The above questions comprised the Orienteering Experience Segment on the administered online questionnaire used in this study.
(PDF)

S1 Table Summarized responses to the orienteering experience questionnaire. Bolded values indicate largest percentage within group.
(PDF)

S1 Data (CSV)

S1 Dataset (CSV)
