# Supplementary material for: Orienteering experts report more proficient spatial processing and memory across adulthood
Source: PLoS One. 2023 Jan 20;18(1):e0280435. doi: 10.1371/journal.pone.0280435 (PMC9858405; doi:10.1371/journal.pone.0280435)
Supplement: S1 Appendix — The above questions comprised the Orienteering Experience Segment on the administered online questionnaire used in this study. (PDF) [file pone.0280435.s001.pdf]

S1 Appendix. Orienteering Experience Questionnaire

Orienteering Experience Segment
The following questions will ask you about your experience in the sport of orienteering. It is STRONGLY encouraged that you answer all questions as this will provide us with the best data for our research. However, only some of the questions are mandatory and you may choose "prefer not to answer" for questions you are not comfortable answering. DO NOT leave a question blank. Please answer questions truthfully and honestly. Remember, you may choose to withdraw yourself from this study at any time. You will be entered into the draw for incentives at any time should you choose to withdraw.

Note: All Mandatory; Questionnaire will not be able to continue if a question is left blank. If participant does not want to answer a non-mandatory question, MUST choose "Prefer not to answer" and CANNOT leave the question blank.)

1. For how many years have you participated in, or did you participate in, the sport of orienteering (at any level)? (mandatory)
    a. 0-5 years
    b. 6-10 years
    c. 11-15 years
    d. 16-20 years
    e. 20+ years
2. What would you consider your skill level in orienteering to be?
    a. Novice (I have trouble navigating on courses that are primarily on trails)
    b. Beginner (I can complete a course that is primarily on trails with no large issues)
    c. Intermediate (I can navigate well both on and off trails, but might have some large issues)
    d. Advanced (I can navigate well off trails with few available features, and seldom make large mistakes)
    e. Elite (I hardly ever make errors in my navigation and feel confident in many kinds of terrains.)
    f. Prefer not to answer
3. On average, how many times per week do you **train, or did you train,** in the sport of orienteering (this does **not** include other sports aside from orienteering)?
    a. 0 times per week
    b. 1-2 times per week
    c. 3-4 times per week
    d. 5-6 times per week
    e. 7 times per week
    f. Prefer not to answer
4. On average, how frequently on a monthly basis do you **race, or did you race,** (at any level, including weeknight C meets, national level A meets and international

competitions, etc) in the sport of orienteering (this does **not** include other sports aside from orienteering)?

   a. 0 – 2 times per month
   b. 3-5 times per month
   c. 6-10 times per month
   d. 11-15 times per month
   e. 16+ times per month
   f. Prefer not to answer

5. On average, how much time do you spend on a weekly basis reading or studying orienteering maps **without** being physically active (ie, orienteering video games, map study, or map making)
   a. hours
   b. 2-4 hours
   c. 5-7 hours
   d. 8-10 hours
   e. 11+ hours
   f. Prefer not to answer
6. Are you a member of a national team in orienteering (Junior, Senior, Masters, etc)?
   a. No, never
   b. No, was previously in the last 0-5 years
   c. No, was previously in the last 6-10 years
   d. No, was previously in the last 11+ years
   e. Yes, currently.
   f. Prefer not to answer
7. What is, or was, your highest race competition level?
   a. I have never raced orienteering.
   b. Club-wide
   c. Provincial wide (or local equivalent)
   d. National level
   e. International level
   f. Prefer not to answer
8. Please check all of the race distances that you have participated in previously (at any level)
   a. Sprint Distance
   b. Sprint relay
   c. Middle Distance
   d. Long Distance
   e. Ultra-Long Distance
   f. Forest Relay
   g. Rogaine
   h. Other: Please list ____________________________
   i. Prefer not to answer
9. Do you use a compass when you participate in the sport of orienteering?

a. Yes, always
b. Sometimes
c. No, never
d. Prefer not to answer

10. Have you ever participated in an Adventure Race (Trekking, paddling, mountain biking) **with navigation** (ie, majority of the race has no marked route)?
    a. No, never
    b. Yes, 1-2 times
    c. Yes, 3-5 times
    d. Yes, 6-10 times
    e. Yes, 11+ times
    f. Prefer not to answer
11. Do you use a map and/or compass outside of orienteering in your daily life (ie, for work)? (ie, **NOT** a self-guiding GPS or app)?
    a. Yes, daily
    b. Yes, frequently (1-2 times per week)
    c. Yes, often (3-5 times per month)
    d. Yes, rarely (1-2 times per month)
    e. No, never
    f. Prefer not to answer
