# Supplementary material for: Orienteering experts report more proficient spatial processing and memory across adulthood
Source: PLoS One. 2023 Jan 20;18(1):e0280435. doi: 10.1371/journal.pone.0280435 (PMC9858405; doi:10.1371/journal.pone.0280435)
Supplement: S1 Table — Bolded values indicate largest percentage within group. (PDF) [file pone.0280435.s002.pdf]

S1 Table. Summarized Responses to the Orienteering Experience Questionnaire

	Intermediate	*Advanced*	*Elite*
n	42	41	31
Q1. Years of Orienteering Experience (%)			
0-5 years	24%	10%	0%
6-10	**29%**	17%	6%
11-15	19%	12%	26%
16-20	12%	15%	19%
20+	17%	**46%**	**48%**
Q3. Weekly Orienteering Training Frequency (%)			
0 times per week	**55%**	12%	0%
1-2 times per week	45%	**59%**	**61%**
3-4 times per week	0%	27%	26%
5-6 times per week	0%	2%	0%
7 times per week	0%	0%	13%
Q4. Monthly Orienteering Racing Frequency (%)			
0-2 times per month	**64%**	39%	6%
3-5 times per month	36%	**44%**	**74%**
6-10 times per month	0%	12%	19%
11-15 times per month	0%	5%	0%
Q5. Weekly Virtual Reality, Mapping or Sedentary Map Study (%)			
0-1 hours	**93%**	**76%**	**65%**
2-4 hours	7%	20%	35%
5-7 hours	0%	5%	0%

Q6. National Team Membership (%)			
No, never	**93%**	**80%**	26%
Previously	5%	12%	29%
Yes, currently	2%	7%	**45%**
Q7. Highest Competition Level (%)			
< International	**88%**	**61%**	16%
International	12%	39%	**84%**
Q8. Participation in Orienteering Disciplines (% Endorsed)			
Sprint	79%	88%	100%
Middle	90%	100%	97%
Long	60%	95%	97%
Forest Relay	7%	54%	87%
Sprint Relay	21%	51%	84%
Ultra-Long	7%	37%	71%
Rogaine	38%	66%	32%
Q9. Use of Compass (%)			
Sometimes or never	29%	10%	3%
Yes, always	**71%**	**90%**	**97%**
Q10. Adventure Race Participation (%)			
No, never	**69%**	**61%**	**65%**
Yes, at least once	31%	39%	35%
Q11. Map and Compass Use Outside of Orienteering (i.e., Work, etc.) (%)			
No, never	38%	24%	29%
Yes, daily or weekly	0%	17%	10%
Yes, monthly	**62%**	**59%**	**61%**
